# A comparison of outcomes between L3/L4 and L4/ L5 single-level laminectomy surgery

**Published:** 2012-11

**Authors:** Parisa Azimi, Hasan Reza Mohammadi

**Affiliations:** ^*a*^Department of Neurosurgery, Shahid Beheshti University of Medical Sciences, Tehran, Iran.

## Abstract

**Background:**

Decompressive laminectomy is the most common operation performed to treat spinal stenosis. This study was performed in order to evaluate surgical outcomes between laminectomy at the L3/L4 level compared with L4/ L5.

**Methods:**

The patients diagnosed with one level stenosis at L3/L4 or L4/ L5 who were candidate for surgery entered into this cross-sectional study. The outcome measures were the Neurogenic Claudication Outcome Score (NCOS), the Japanese Orthopaedic Association (JOA) Score, subjective walking distance, and Visual Analog Score (VAS) of leg pain/numbness. T-test tests were used to analyze the comparisons of outcomes between the L3/L4 and L4/ L5 single-level laminectomy.

**Results:**

Ninety-four patients were eligible to enter the study during the four- year courses of study. Patients were aged 39 to 79 years (mean age 63.7 ± 9.83 years) and were followed up for at least one year. Thirty-one L3/4 and 63 L4/L5 laminectomies were performed. No significant difference was observed in the clinical indications, JOA, NCOS, duration of symptoms or VAS of leg pain/numbness between the two groups. The difference between pre and postoperative was statistically significant (P less than 0.0001).

**Conclusions:**

The findings of this study suggest that no statistically signiﬁcant difference exists between L3/L4 and L4/ L5 laminectomies in terms of preoperative and postoperative outcomes.

**Keywords:**

Spinal stenosis, Laminectomy, Single-level, Outcome measures


					Volume 4, Suppl. 1 Nov 2012
Publisher: Kermanshah University of Medical Sciences
URL: http://www.jivresearch.org
Copyright:
Congress of Iranian Neurosurgeons, 14-16 November 2012, Kermanshah, Iran
Paper No. 64
A comparison of outcomes between L3/L4 and L4/ L5
single-level laminectomy surgery
Parisa Azimi a,*
, Hasan Reza Mohammadi a
a
Department of Neurosurgery, Shahid Beheshti University of Medical Sciences, Tehran, Iran.
Abstract:
Background: Decompressive laminectomy is the most common operation performed to treat spinal stenosis. This study
was performed in order to evaluate surgical outcomes between laminectomy at the L3/L4 level compared with L4/
L5.
Methods: The patients diagnosed with one level stenosis at L3/L4 or L4/ L5 who were candidate for surgery entered
into this cross-sectional study. The outcome measures were the Neurogenic Claudication Outcome Score (NCOS), the
Japanese Orthopaedic Association (JOA) Score, subjective walking distance, and Visual Analog Score (VAS) of leg
pain/numbness. T-test tests were used to analyze the comparisons of outcomes between the L3/L4 and L4/ L5 single-
level laminectomy.
Results: Ninety-four patients were eligible to enter the study during the four- year courses of study. Patients were
aged 39 to 79 years (mean age 63.7  9.83 years) and were followed up for at least one year. Thirty-one L3/4
and 63 L4/L5 laminectomies were performed. No significant difference was observed in the clinical indications, JOA,
NCOS, duration of symptoms or VAS of leg pain/numbness between the two groups. The difference between pre
and postoperative was statistically significant (P less than 0.0001).
Conclusions: The findings of this study suggest that no statistically significant difference exists between L3/L4 and
L4/ L5 laminectomies in terms of preoperative and postoperative outcomes.
Keywords:
Spinal stenosis, Laminectomy, Single-level, Outcome measures
* Corresponding Author at:
Parisa Azimi: Department of Neurosurgery, Imam-Hossain Hospital, Shahid-Beheshti University of Medical Sciences, Imam-Hossain sq., Tehran, Iran.
Email: Parisa.azimi@gmail.com, (Azimi P.).

				

